# Vivisection Indispensable to the Progress of Medicine

**Published:** 1885-09

**Authors:** 


### Vivisection Indispensable to the Progress of Medicine.

Wounds of the abdomen, especially gunshot wounds, are among the most fatal injuries known to surgery. A small, innocent-looking external pistol wound may cover multiple and almost inevitably fatal perforations of the abdominal contents. The recoveries from 3,717 such wounds during the late civil war only numbered 444, and of those with escape of the intestinal contents the recoveries, says Otis, may be counted on one's fingers. The prevailing treatment, as laid down in our textbooks, has been purely conservative, treating symptoms as they arise. The brilliant results achieved in other abdominal operations have led a few bold spirits, such as our own Sims, Gross, Otis, McGuire and others, to advocate the opening of the abdomen and the repair of the injuries found.

In May of last year, Parkes, of Chicago, reported to the American Medical Association a series of systematic experiments on 37 dogs that were etherized, then shot, the abdomen opened, and the wounds of the intestines, arteries, mesentery, etc., treated by appropriate surgical methods. The results confirmed the belief awakened by earlier experiments and observations that surgery could grapple successfully with multiple and formidable wounds, by sewing them up in various ways, or even by removing a piece of the bowel and uniting the cut ends. Hard upon the heels of this important paper, and largely as its result, comes a striking improvement in practice. And, remember, that this is only the first fruit of a rich harvest for future time, in all countries in peace and in war.

November 2d, of last year, a man was brought to the Chambers Street Hospital, in New York, with a pistol shot wound in the abdomen. Under careful antiseptic precautions, and following the indications of Parkes, the abdomen was opened by Dr. Bull, coil after coil of the intestines was drawn out, the bullet was found and removed, and seven wounds cf the intestines were successively discovered and properly treated, and the patient made an uninterrupted recovery. A recovery, after so many wounds, any one of which would necessarily have been fatal under the old methods of treatment, shows that we have now entered upon a proper and successful method of treatment for such frightful accidents.

